# The Importance of Integrated Healthcare in the Association Between Oral Health and Awareness of Periodontitis and Diabetes in Type 2 Diabetics

**DOI:** 10.3290/j.ohpd.b875369

**Published:** 2021-01-26

**Authors:** Flavia Bridi Valentim, Vinícius Cavalcanti Carneiro, Patrícia da Costa Gomes, Elizabeth Pimentel Rosetti

**Affiliations:** a Master in Dental Clinics, Department of Dental Prosthesis, Federal University of Espírito Santo, Vitória, Espírito Santo, Brazil. Idea, hypothesis, experimental design, wrote the manuscript, proofread the manuscript and performed statistical evaluation.; b Master in Dental Clinics, Department of Dental Prosthesis, Federal University of Espírito Santo, Vitória, Espírito Santo, Brazil. Performed the experiments and proofread the manuscript.; c Master in Dental Clinics, Department of Dental Prosthesis, Federal University of Espírito Santo, Vitória, Espírito Santo, Brazil. Performed the experiments and proofread the manuscript.; d Master in Dentistry and Professor, Department of Dental Prosthesis, Federal University of Espírito Santo, Vitória, Espírito Santo, Brazil. idea, hypothesis, proofread the manuscript, performed statistical evaluation and contributed substantially to discussion.

**Keywords:** diabetes mellitus, periodontal diseases, health promotion, public health/community dentistry, primary healthcare

## Abstract

**Purpose:**

To assess the association of various factors including education level and oral health with type 2 diabetics’ awareness of periodontitis and periodontitis/diabetes relationship, and to evaluate the importance of integrated healthcare in this association.

**Materials and Methods:**

288 type 2 diabetics were evaluated through a validated structured questionnaire about oral hygiene habits, access and attendance to dental treatment, the presence of periodontitis and previously received information of periodontitis and periodontitis/diabetes relationship. Descriptive data were explored and both simple and multiple logistic regressions were performed.

**Results:**

The average age of participants was 62.24 (±10.93) years, 81.6% were previously treated for periodontitis and approximately 70% have never received information on periodontitis and its relationship with diabetes. A higher chance of participants having previously received information regarding periodontitis was associated with more than 8 years of schooling, daily flossing habit, presence of periodontitis and prior treatment for periodontitis (p < 0.005). Regarding previously received information about periodontitis/diabetes relationship, statistically significant associations were observed for more than 12 years of schooling and diabetes diagnosed more than 8 years ago (p < 0.05).

**Conclusion:**

The vast majority of participants were previously treated for periodontitis without receiving proper oral health education, which means that access to costly dental treatment is provided while patient education is neglected. It was shown the influence of habits and living conditions on the previously received information about diseases, and therefore, particular attention to the population characteristics is important to make the information accessible to everyone.

The International Diabetes Federation reported 425 million diabetics over the world in 2017, which represented 8.8% of the adult population.^17^ The World Health Organization estimates that diabetes mellitus will figure as the seventh leading cause of death in 2030. Type 2 diabetes accounts for at least 90% of all cases.^31^

Diabetes and periodontitis are highly prevalent chronic diseases with similar pathobiology and a proven bidirectional relationship.^6^ Uncontrolled (or poorly controlled) diabetes increases the risk, extent, and severity of periodontitis,^5,22,24^ while advanced periodontitis also compromises glycaemic control.^5,22^

It is generally accepted that periodontitis is more prevalent and severe in diabetics; thus, periodontal signs and symptoms are recognised as the ‘sixth complication’ of diabetes.^21^ The link between periodontitis and altered glycaemic control is still unclear, but it is believed that proinflammatory mediators expressed by periodontal sites enter the systemic circulation and affect the normal function of insulin receptors (suppression of signalling process), which in turn contributes to increased insulin resistance and impaired of glucose homeostasis.^12^

In this scenario, various studies have demonstrated the importance of oral diseases control to treat and prevent diabetes since both conventional and surgical periodontal therapies have led to the reduction of glycated haemoglobin (HbA1c) levels.^10,15,28^ A better understanding of the relationship between periodontitis and systemic diseases has the potential to economically influence healthcare policies since 12% of the global expenditures on healthcare are directed to the treatment of diabetes and related complications.^16,17^ The substantial cost of diabetes treatment remains a statistically significant challenge for healthcare systems and an obstacle for a sustainable economic development.^17^

Since the guidance and motivation of patients are important factors for the success of periodontal disease treatment,^9^ comprehensive oral health policies for diabetics are needed. Therefore, better collaboration between physicians and dentists (medical care team) may result in more effective clinical practices.^11^

Thus far, little is known about the patients’ knowledge about diabetes and its relationship with periodontal health. Only a few studies have recently investigated attitudes, oral health knowledge and behaviours among diabetics.^2,3,8,13^ Therefore, the aims of this study were (1) to assess the association of various factors including education level and oral health with type 2 diabetics’ awareness of periodontitis and periodontitis/diabetes relationship, and (2) to evaluate the importance of integrated healthcare at a Brazilian public healthcare system.

## Materials and Methods

### Ethical Approval

This study was conducted according to the Helsinki Declaration and approved by the Research Ethics Committee of the Federal University of Espírito Santo under file number 1,749,053. All subjects voluntarily signed the informed consent form.

### Study Design

A total of 288 males and females (aged 18 years older) with at least one tooth, diagnosed with type 2 diabetes and registered on a hypertension and diabetes control programme promoted by three public healthcare basic units in Vitória (capital of Espírito Santo state, Brazil), were selected to participate in this cross-sectional epidemiologic survey. Smokers were excluded due to scientific evidence of a causal relationship between tobacco and periodontal disease.^14,30^ In order to provide a representative sample of the municipality, the healthcare basic units were selected from three health districts with different sociodemographic profiles by means of block-permuted randomisation. The sample was stratified by proportional allocation in order well represent each unit. By using the Action Stat software (Estatcamp, Brazil), which is developed on a free programming language named R (R Core Team, Austria), the sample size was calculated based on 1,200 diabetics registered in the three selected units by using a statistical test of a simple random sample with a confidence level of 95% and 5% as the maximum expected error.

### Data Collection

Data was collected through a validated structured questionnaire^7^ applied by a previously trained examiner. The subjects were invited to participate in the survey while waiting for a medical appointment and time to respond ranged from 5 to 10 min. Questions included demographic and economic aspects, oral hygiene habits, access and attendance to dental treatment, the presence of periodontitis, and previously received information regarding periodontitis and periodontitis/diabetes relationship. The presence of periodontitis was based on questions about tooth mobility and migration, gingival recession, tooth loss without professional extraction and bone loss. In case of at least one positive response, the subject was considered to have periodontitis.^18,19^ Then, participants received a guidance booklet about diabetes, periodontal disease, and their bidirectional relationship. Subjects who did not agree to participate or returned incomplete questionnaires were replaced.

### Statistical Analysis

A statistical software (IBM SPSS Statistics v24, IBM Corp) was used to explore descriptive data (means, standard deviation, and percentage frequency). Simple and multiple logistic regressions with a statistical significance level of 5% and 95% confidence interval were used to verify whether the variables (education level, period of time since diabetes was diagnosed, toothbrushing frequency, flossing frequency, presence of periodontitis and prior treatment for periodontitis) are associated with participants’ awareness of periodontitis and periodontitis/diabetes relationship.

## Results

### Sample Characterisation

The average age was 62.24 (±10.93) years, 62% of participants were women, more than half of the sample declared up to 8 years of schooling, monthly household income up to three minimum wages was observed for 74% of participants, and 61% were diagnosed with diabetes less than 8 years ago (Table 1).

**Table 1:** Characterisation of the sample, oral healthcare, and received information

Age (mean ± standard deviation)	62.24 (±10.93)		
Age (median/minimum-maximum)	63 (28–93)		
		n	%
Gender	Male	107	37.15
	Female	181	62.85
Education level	<8 years	155	53.82
	8–12 years	86	29.86
	>12 years	47	16.32
Monthly household income (minimum wages^*^)	1–3	215*	74.65
	3–5	34	11.81
	>5	39	13.54
Period of time since diabetes was diagnosed	≤8 years	176	61.11
	>8 years	112	38.89
Toothbrushing frequency	No brushing	0	0.00
	Once per day	17	5.9
	Twice per day	81	28.13
	≥3 times per day	190	65.97
Flossing frequency	No flossing	125	43.4
	1–3 times per week	37	12.85
	Daily	126	43.75
Period of time since last dental appointment (check-up or treatment)	Up to 1 year	190	65.97
	>1 year	98	34.03
Medical referral for dental treatment	No	248	86.11
	Yes	40	13.89
Presence of periodontitis	No	122	42.36
	Yes	166	57.64
Previous periodontitis treatment	No	53	18.4
	Yes	235	81.6
Have you ever received information regarding periodontitis?	No	199	69.1
	Yes	89	30.9
Have you ever received information on the relationship between periodontitis and diabetes?	No	224	77.78
	Yes	64	22.22

^*^1 minimum wage ≈ U$290.

More than 80% of the participants were previously treated for periodontitis; 69.1% and 77.78% have never received information on periodontitis and its relationship with diabetes, respectively (Table 1).

### Association with Periodontitis Awareness

Both simple and multiple logistic regressions associated participants with more than 12 years of schooling, daily flossing habit and presence of periodontitis with a higher chance of having previously received information regarding periodontitis. This association was also indicated by simple logistic regression in cases of more than 8 years of schooling and prior treatment for periodontitis (p <0.005) (Table 2).

**Table 2:** Association between received information regarding periodontitis and education level, the period of time since diabetes was diagnosed, oral hygiene, presence and treatment of periodontitis. A higher OR is indicating a higher chance that the participants had received information

		Have you ever received information regarding periodontitis?				OR (gross^**^)	p value	OR (adjusted^***^)	p value
		No		Yes^*^					
		n	%	n	%				
Education level	<8 years	122	63.54	33	34.38	1	–	1	–
	8–12 years	52	27.08	34	35.42	2.41 (1.35–4.31)	**0.003**	1.72 (0.90–3.25)	0.096
	>12 years	18	9.38	29	30.21	5.95 (2.95–12.0)	**< 0.001**	3.96 (1.82–8.59)	**< 0.001**
Period of time since diabetes was diagnosed	≤8 years	120	62.50	56	58.33	1	–	1	–
	>8 years	72	37.50	40	41.67	1.19 (0.72–1.96)	0.494	1.17 (0.66–2.08)	0.593
Toothbrushing frequency	Once per day	13	6.77	4	4.17	1	–	1	–
	Twice per day	61	31.77	20	20.83	1.06 (0.31–3.64)	0.919	0.84 (0.21–3.30)	0.809
	≥3 times per day	118	61.46	72	75.00	1.44 (0.39–5.26)	0.247	0.99 (0.26–3.72)	0.989
Flossing frequency	None	98	51.04	27	28.13	1	–	1	–
	1–3 times per week	30	15.63	7	7.29	0.84 (0.33–2.13)	0.725	0.72 (0.26–1.97)	0.528
	Daily	64	33.33	62	64.58	3.51 (2.02–6.09)	**< 0.001**	2.61 (1.37–4.98)	**0.003**
Presence of periodontitis	No	116	60.42	34	35.42	1	–	1	–
	Yes	76	39.58	62	64.58	2.78 (1.67–4.62)	**< 0.001**	2.55 (1.45–4.47)	**0.001**
Previous periodontitis treatment	No	41	21.35	7	7.29	1	–	1	–
	Yes	151	78.65	89	92.71	3.45 (1.48–8.02)	**0.004**	2.24 (0.89–5.59)	0.084

n: number; %: percentage; OR: odds ratio; ^*^Set as reference category in the regression analysis; ^**^Simple logistic regression; ^***^Multiple logistic regression adjusted to all variables.

### Association with periodontitis/diabetes relationship awareness

A higher chance of participants having previously received information on the relationship between periodontitis and diabetes was associated with more than 12 years of schooling (by means of simple logistic regression) and diabetes diagnosed more than 8 years ago (by means of multiple logistic regression) (p <0.05) (Table 3).

**Table 3:** Association between received information on the periodontitis/diabetes relationship and education level, the period of time since diabetes was diagnosed, oral hygiene, presence and treatment of periodontitis. A higher OR is indicating a higher chance that the participants had received information

		Have you ever received information about the relationship between periodontitis and diabetes?				OR (gross^**^)	p value	OR (adjusted^***^)	p value
		No		Yes^*^					
		n	%	n	%				
Education level	<8 years	124	55.86	31	46.97	1	–	1	–
	8–12 years	67	30.18	19	28.79	1.13 (0.81–3.09)	0.701	1.08 (0.54–2.14)	0.828
	>12 years	31	13.96	16	24.24	2.06 (1.0–4.24)	0.049	1.74 (0.80–3.80)	0.159
Period of time since diabetes was diagnosed	≤8 years	145	65.32	31	46.97	1	–	1	–
	>8 years	77	34.68	35	53.03	2.12 (1.21–3.71)	0.008	2.15 (1.21–3.82)	0.008
Toothbrushing frequency	Once per day	14	6.31	3	4.55	1	–	1	–
	Twice per day	63	28.38	18	27.27	1.33 (0.34–5.15)	0.677	1.47 (0.35–6.06)	0.591
	≥3 times per day	145	65.32	45	68.18	1.44 (0.39–5.26)	0.574	1.42 (0.35–5.69)	0.619
Flossing frequency	None	99	44.59	26	39.39	1	–	1	–
	1–3 times per week	28	12.61	9	13.64	1.22 (0.51–2.91)	0.648	1.15 (0.46–2.84)	0.758
	Daily	95	42.79	31	46.97	1.24 (0.68–2.24)	0.472	1.10 (0.55–2.18)	0.785
Presence of periodontitis	No	121	54.50	29	31.82	1	–	1	–
	Yes	101	45.50	37	68.18	1.52 (0.87–2.65)	0.133	1.35 (0.76–2.40)	0.303
Previous periodontitis treatment	No	40	18.02	8	12.12	1	–	1	–
	Yes	182	81.98	58	87.88	1.59 (0.70–3.59)	0.262	1.47 (0.62–3.48)	0.377

n: number; %: percentage; OR: odds ratio; ^*^Set as reference category in the regression analysis; ^**^Simple logistic regression; ^***^Multiple logistic regression adjusted to all variables.

## Discussion

The null hypothesis tested was that participants’ awareness of periodontitis and periodontitis/diabetes relationship are not associated with education level, the period of time since diabetes was diagnosed, hygiene and oral health habits. Statistically significant associations between some of the predictor variables and the response variable were found; thus, the null hypothesis had to be partially rejected.

The highest education level was associated with a higher chance of participants having previously received information on periodontitis and periodontitis/diabetes (p <0.05), which can be explained by the amount of knowledge acquired during more years of study. Bahamman^2^ also observed a statistically significant association between education level and knowledge about periodontitis/diabetes relationship.

Education should ideally be accessible to everyone, thus healthcare professionals must give special attention to people who do not have access to other means of guidance and provide accurate and high-quality information on their health. The means through which participants were informed about periodontitis and its association with diabetes were not investigated; however, Habashneh et al,^13^ Bahmmam^2^ and Eldarrat^8^ reported that diabetic patients were informed through television, internet, magazines, school, family, friends, and by health services.

Participants who reported to floss their teeth on a daily basis, classified with periodontitis and previously treated for periodontitis presented higher chance of having previously being educated on what periodontal disease is, probably due to regular dental appointments and more care with their own oral health.^9^ The fact that only a small part in our study sample was aware of periodontitis and its relationship with diabetes corroborates with Habashneh et al,^13^ Strauss et al,^27^ Umeizudike et al^29^ and Yuen et al,^32^ which observed the lack of proper patient guidance often associated with limited oral health knowledge of healthcare professionals.

Although many patients reported that they received insufficient information about the investigated diseases, the vast majority of participants were previously treated for periodontitis. Therefore, it seems clear that access to dental treatment is not the issue, but rather providing information on periodontitis and diabetes to patients, which is much cheaper and comprehensive.

In order to better disseminate information within diabetics, it is suggested that healthcare professionals adopt ongoing proceedings of knowledge transmission and measures for patients’ motivation. The empowerment of each patient by following a daily at-home oral care routine and not solely dependent on treatments provided at dental offices is a key factor for the maintenance of a healthy periodontium.

Another relevant aspect of this study is that most of the participants are female, as well as the investigations conducted by Pathak et al^23^ and Silva et al.^26^ Some studies have reported that women consult their general physicians more frequently and take more medications than men, which suggests greater difficulty in controlling diabetes in females.^1,13^ In this line, healthcare driven by gender becomes relevant.

Potential limitations may be related to the validity of the collected data since self-reports were not validated by intraoral examination. In addition, participants with advanced age may not always recollect previously received information.^4^

Studies such as this become important to understand the level of diabetics’ awareness on their own health (oral hygiene habits, the importance of regularly attend medical and dental appointments, bidirectional relationship between and periodontitis and diabetes) and provide crucial information to healthcare system planning, allocation of resources and determination of work guidelines for healthcare professionals.^20,25^ Moreover, this is one of the few studies in current literature that addresses the association of various factors (education level, the period of time since diabetes was diagnosed, oral health and history of dental treatment) with diabetics’ received information on periodontal disease and its relationship with glycaemic control.

## Conclusion

The vast majority of participants were previously treated for periodontitis without receiving proper oral health education, which means that access to costly dental treatment is provided while a practical and inexpensive education for patients is underestimated. It was shown the influence of habits and living conditions on the previously received information about diseases, and therefore, healthcare professionals must be aware of the population characteristics to make the information accessible to everyone.
